# Intravenous Administration of Remdesivir at the Acute Phase of SARS-CoV-2 Infection Is Associated with a Lower Prevalence of Post-COVID-19 Pain

**DOI:** 10.3390/jcm14093156

**Published:** 2025-05-02

**Authors:** César Fernández-de-las-Peñas, Anabel Franco-Moreno, María Ruiz-Ruigómez, Estibaliz Arrieta-Ortubay, Pablo Ryan-Murua, Carlos Lumbreras-Bermejo, Pablo del-Valle-Loarte, Oscar J. Pellicer-Valero, Juan Torres-Macho, Rocco Giordano, Lars Arendt-Nielsen

**Affiliations:** 1 Department of Physical Therapy, Occupational Therapy, Physical Medicine and Rehabilitation, Universidad Rey Juan Carlos, 28922 Madrid, Spain; 2 Center for Neuroplasticity and Pain (CNAP), Sensory-Motor Interaction (SMI) Center, Department of Health Science and Technology, Faculty of Medicine, Aalborg University, 9220 Aalborg, Denmark; rg@hst.aau.dk (R.G.); lan@hst.aau.dk (L.A.-N.); 3 Department of Internal Medicine, Hospital Universitario Infanta Leonor-Virgen de la Torre, 28031 Madrid, Spain; anaisabel.franco@salud.madrid.org (A.F.-M.); pabloryan@gmail.com (P.R.-M.); juan.torresm@salud.madrid.org (J.T.-M.); 4 Department of Internal Medicine, Hospital Universitario Doce de Octubre, 28041 Madrid, Spain; rryruiz@gmail.com (M.R.-R.); earrietao@gmail.com (E.A.-O.); carlos.lumbreras@salud.madrid.org (C.L.-B.); 5 CIBER de Enfermedades Infecciosas (CIBERINFEC), Instituto de Salud Carlos III (ISCIIII), 28220 Madrid, Spain; 6 Department of Internal Medicine, Hospital Universitario Severo Ochoa, 28911 Madrid, Spain; pablo.valle@salud.madrid.org; 7 Image Processing Laboratory (IPL), Universitat de València, Parc Científic, 46980 Paterna, Spain; oscar.pellicer@uv.es; 8 Department of Medicine, School of Medicine, Universidad Complutense de Madrid, 28040 Madrid, Spain; 9 Department of Oral and Maxillofacial Surgery, Aalborg University Hospital, 9100 Aalborg, Denmark; 10 Department of Gastroenterology & Hepatology, Mech-Sense, Clinical Institute, Aalborg University Hospital, 9100 Aalborg, Denmark; 11 Steno Diabetes Center North Denmark, Clinical Institute, Aalborg University Hospital, 9100 Aalborg, Denmark

**Keywords:** antiviral, Remdesivir, pain, post-COVID-19

## Abstract

**Background/Objective:** Evidence suggests that the administration of antivirals at the acute phase of SARS-CoV-2 infection is associated with lower COVID-19 severity, accordingly, the administration of antivirals at the acute phase of the infection could prevent post-COVID-19 symptoms. The current study investigated the effects of the intravenous administration of Remdesivir at hospitalization (acute phase of SARS-CoV-2 infection) in COVID-19 survivors on the development of post-COVID-19 pain symptoms. **Methods**: A cohort of previously hospitalized COVID-19 survivors who received intravenous administration of Remdesivir at the acute COVID-19 phase (*n* = 216) were matched with a cohort of previously hospitalized COVID-19 survivors who did not receive any antiviral treatment at the acute phase of the infection (*n* = 216). In a face-to-face interview, they were asked for the development of pain symptoms attributed to SARS-CoV-2 infection and whether the symptom persisted at the time of the study (mean follow-up: 18.4, SD: 0.8 months). Clinical/hospitalization data were collected from medical records. Anxiety/depressive symptoms and sleep quality were also assessed with validated self-reported questionnaires. **Results:** No differences in hospitalization data and the presence of previous chronic conditions were seen between patients receiving or not receiving intravenous administration of Remdesivir during hospitalization. The multivariate analysis revealed that the intravenous administration of Remdesivir at the acute COVID-19 phase was a protective factor for the development of overall post-COVID-19 pain (OR 0.444, 95% CI 0.292–0.674, *p* < 0.001). A protective effect of administrating intravenous Remdesivir was specifically seen for thorax/chest (OR 0.277, 95% CI 0.100–0.766, *p* = 0.01) and lumbar spine (OR 0.347, 95% CI 0.143–0.844, *p* = 0.02) pain. **Conclusions**: Current results support a potential protective role of the intravenous administration of Remdesivir at the acute phase of SARS-CoV-2 infection for developing long-term post-COVID-19 pain in previously hospitalized COVID-19 survivors. Studies investigating the effects of the oral administration of antivirals in non-hospitalized populations are needed to generalize these findings.

## 1. Introduction

The rapid spreading of the coronavirus disease 2019 (COVID-19) has resulted in one of the most severe worldwide pandemics of this century. Early intervention with antiviral medications, e.g., Nirmatrelvir-Ritonavir or Remdesivir, during the acute phase of COVID-19 has been linked to lower mortality, lower disease severity, shorter hospital stays, and shorter time until negative swab test results [1]. The primary rationale for administering antivirals during severe acute respiratory syndrome coronavirus 2 (SARS-CoV-2) infection is their ability to inhibit virus replication, thereby preventing the progression to a more severe condition [2]. Remdesivir is a nucleotide analog that acts by binding to the viral RNA-dependent polymerase, causing premature termination of RNA transcription and, consequently, hindering viral replication [3]. The recommended dosage for Remdesivir administration during the acute phase of COVID-19 is 200 mg on the first day, followed by 100 mg/day for five consecutive days, with a maximum duration of 10 days [4].

A new significant healthcare concern associated with COVID-19 is the development or presence of long-lasting symptomatology following acute SARS-CoV-2 infection [5]. These lingering symptoms, referred to as long-COVID [6] or post-COVID-19 condition [7], have been documented in up to 25–30% of subjects who have recovered from SARS-CoV-2 infection, with symptoms persisting for one [8,9] or even two years [10,11]. Given these findings, identifying strategies that mitigate the risk of developing post-COVID-19 symptoms is of great importance [12]. A systematic review assessed the potential protective role of administering an antiviral such as Remdesivir during the acute COVID-19 phase against long-lasting post-COVID-19 symptomatology development, but findings were inconclusive [13]. This review examined three studies evaluating if Remdesivir reduced the risk of post-COVID-19 symptoms in previously hospitalized patients, with two studies supporting a protective effect [14,15] while the third one did not [16]. Thus, a recent study indicated that Remdesivir administration was associated with earlier symptom resolution as reported by the patients at post-infection, but without substantial improvements in functional recovery [17]. These investigations primarily assessed the overall occurrence of post-COVID-19 symptoms without specifically focusing on specific symptoms such as pain [14,15,16,17].

Pain is among the long-lasting symptoms experienced by approximately 15–20% of individuals who have recovered from an acute SARS-CoV-2 infection within the first six months [18]. A recent meta-analysis estimated that the prevalence of post-COVID-19 pain-related symptoms during the first year after SARS-CoV-2 infection was 8% for chest pain, 18% for joint pain, 17% for muscle pain, and 12% for headaches [19]. However, no studies have specifically examined whether administrating Remdesivir during the acute SARS-CoV-2 phase influences the risk of developing post-COVID-19 pain symptoms [14,15,16,17].

This study aimed to evaluate the impact of the intravenous administration of Remdesivir during the acute phase of SARS-CoV-2 infection in hospitalized COVID-19 survivors on the development of post-COVID-19 pain symptoms. Our hypothesis proposed that individuals who received intravenous administration of Remdesivir during hospitalization due to acute COVID-19 would exhibit a lower incidence of post-COVID-19 pain symptoms compared to those who did not receive the antiviral treatment.

## 2. Methods

### 2.1. Participants

Two cohorts of individuals who needed hospitalization due to an acute SARS-CoV-2 infection at four different urban hospitals in Madrid (Spain) between September 2020 and March 2021 were included in this study. The dominant variants of concern circulating during hospitalization time of both cohorts included the historical strain (20A.EU2) from September to December 2020 and the Alpha variant (B.1.1.7) from January to March 2021. SARS-CoV-2 infection was confirmed through reverse transcription–polymerase chain reaction (RT-PCR) testing of nasopharyngeal or oral swab samples, alongside clinical and radiological findings at hospital admission. The study design received approval from the Institutional Ethics Committees of all participating institutions/hospitals (H12OCT23/418 28/07/2020; URJC0907202015920 15/07/2020; HUIL/092-20 6/09/2020; HSO25112020 4/09/2020; HCSC20/495E 10/07/2020). Informed consent was obtained from all participants prior to data collection. Both cohorts selected in this study were hospitalized before (September–December 2020) and at the initial phase of Spain’s COVID-19 vaccination program (January–March 2021). None of the included patients had been vaccinated before their hospitalization due to SARS-CoV-2.

The first cohort comprised COVID-19 survivors receiving the intravenous administration of Remdesivir as an antiviral treatment during their hospitalization for acute COVID-19. The treatment protocol included an initial dose of 200 mg on the first day, followed by 100 mg per day for five consecutive days or until discharge if the hospitalization period was shorter than five days, with a maximum treatment duration of 10 days [4]. The second cohort included in this study was formed by previously hospitalized COVID-19 survivors who did not receive any antiviral treatment during the acute phase of infection at the hospital. These individuals were matched with the first cohort based on age, sex, body mass index, and vaccination status (since none had received any vaccine dose before the infection).

### 2.2. Data Collection

Demographic data (age, gender, height, and weight), clinical history (pre-existing chronic pain conditions and vaccination status), and hospitalization details (intensive care unit—ICU—admission, duration of hospital stay, and whether Remdesivir was intravenously administered) were gathered from the hospital medical records for both cohorts. Special consideration was given to any pre-existing medical conditions, such as arthritis or migraines, that could contribute to the development of post-COVID-19 pain symptomatology.

Participants who consented to take part in the study were scheduled for a telephone interview conducted by trained healthcare researchers who were blinded to the cohort. A specific questionnaire assessing post-COVID-19 pain symptomatology was developed for the study. Chronic musculoskeletal pain was defined based on the criteria set by the International Association for the Study of Pain [20]. Participants were asked whether they had experienced pain that emerged following their hospitalization due to COVID-19, persisted for at least three consecutive months, and was still present at the time of the study. Additionally, they were required to specify the location of their pain symptoms, including areas such as the head, neck, shoulder, elbow–wrist, hip, knee, thorax, lower extremity, upper extremity, or generalized pain.

Given the strong correlation between pain and mood disorders, the Hospital Anxiety and Depression Scale (HADS) [21] and the Pittsburgh Sleep Quality Index (PSQI) [22] were administered to evaluate anxiety/depression symptoms and sleep quality, respectively. Both questionnaires are validated for telephone administration [23]. HADS includes separate scales for anxiety (HADS-A, 7 items, 0–21 points) and depressive (HADS-D, 7 items, 0–21 points) levels. HADS has demonstrated strong validity and reliability among individuals with long-COVID [24]. A score of ≥8 points on either scale was used to identify significant anxiety or depressive symptoms, based on established sensitivity and specificity thresholds [25]. PSQI (0–21 points) measures sleep quality over the past month, with a cut-off score of ≥8 points indicating poor sleep quality [22].

### 2.3. Statistical Analysis

Data collection was conducted using STATA 16.1, while data processing and analysis were performed with Python’s statsmodels 0.13.2 library. Scipy 1.7.3 was utilized for statistical testing, and statsmodels 0.11.0 was employed for *p*-value correction. Categorical data were reported as the number of cases (percentage), whereas quantitative data were presented as mean and standard deviation (SD). Comparisons of post-COVID-19 pain symptom development between COVID-19 survivors who received the intravenous administration of Remdesivir and those who did not were carried out by using Chi-squared or ANOVA tests, depending on the nature of the data. The significance level was set at 0.05, with *p*-values adjusted using the Holm–Bonferroni correction. Additionally, adjusted odds ratios (ORs) and 95% confidence intervals (CIs) were calculated through multivariate logistic regression models with all pre-existing conditions included into the analysis to assess the risk of developing post-COVID-19 pain symptoms. In the multivariate analysis, the significance level was pre-determined at 0.05, as no Type I error correction was applied.

## 3. Results

A total of 300 previously hospitalized patients (75 randomly selected from each hospital) who had received intravenous Remdesivir during the acute phase of infection were invited to participate. Of these, 216 individuals (mean age: 55.4 years old, SD: 12.6; 43.5% women) agreed to take part in the study. A matched cohort consisting of 216 previously hospitalized COVID-19 survivors who did not receive any antiviral treatment during the acute infection phase at hospitalization was also included. This cohort was matched based on sex, age, body mass index (BMI), and vaccination status (mean age: 55.6 years old, SD: 12.7; 43.5% women).

No significant differences were observed in hospitalization duration or ICU admission between patients who received intravenous administration of Remdesivir and those who did not (Table 1). Similarly, no differences were found regarding pre-existing chronic pain conditions between both groups (Table 1). Participants who received Remdesivir were assessed 18.3 months (SD: 0.7) post-hospitalization, whereas those who did not receive antiviral treatment were assessed after 18.4 months (SD: 1.0) (*p* = 0.8). At the time of the study, the prevalence of long-lasting post-COVID-19 pain was 22.7% (*n* = 49) among individuals treated with Remdesivir during hospitalization and 39.8% (*n* = 86) in those who did not receive the antiviral treatment (OR 0.444, 95% CI 0.292–0.674, *p* < 0.001).

**Figure 1:**
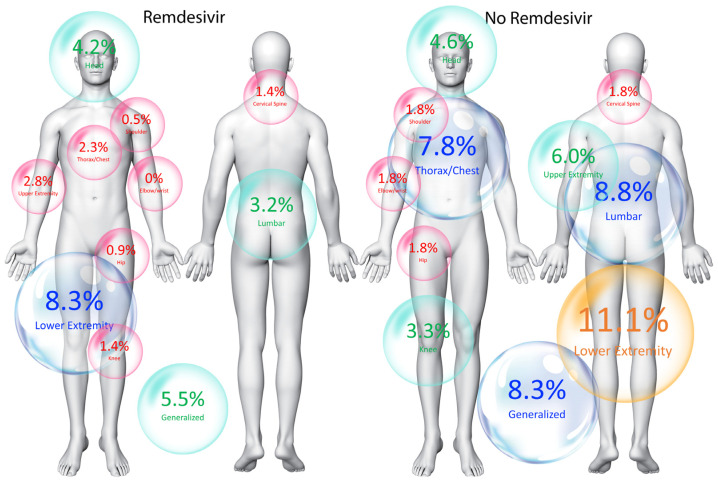
Location of post-COVID-19 pain symptoms individuals who received intravenous administration of Remdesivir at acute COVID-19 phase (**left side**) and those who did not (**right side**) at six months after hospital discharge.

Overall, the locations of post-COVID-19 pain symptoms were similar between patients who received intravenous administration of Remdesivir at hospitalization and those who did not (Figure 1). The only difference was that patients who received Remdesivir at the acute phase reported a lower prevalence rate of post-COVID-19 pain in the thorax/chest and in the lumbar spine (both, *p* < 0.02) than those who did not receive antiviral treatment (Table 1). The multivariate analysis revealed a protective factor of intravenous administration of Remdesivir for pain in the thorax/chest (OR 0.277, 95% CI 0.100 to 0.766, *p* = 0.01) and in the lumbar spine (OR 0.347, 95% CI 0.143 to 0.844, *p* = 0.02).

Additionally, a significant (*p* < 0.001) higher proportion of patients not receiving antiviral treatment reported anxiety (*n* = 70, 32.4%) and depressive (*n* = 80, 37.05%) symptoms when compared with those receiving intravenous administration of Remdesivir at the acute phase (*n* = 4, 1.85% anxiety, *n* = 7, 3.2% depression). No significant differences (*p* = 0.935) in the presence of poor sleepers were found between patients who received Remdesivir (*n* = 77, 35.6%) and those who did not (*n* = 76, 35.3%). Accordingly, the intravenous administration of Remdesivir at the acute COVID-19 phase was a protective factor for self-reporting post-COVID-19 anxiety (OR 0.039, 95% CI 0.014 to 0.110, *p* < 0.001) and depressive (OR 0.057, 95% CI 0.026 to 0.127, *p* < 0.001) symptomatology, but not poor sleep (OR 1.020, 95% CI 0.688 to 1.514, *p* = 0.920).

## 4. Discussion

The current study observed that previously hospitalized COVID-19 survivors who received the intravenous administration of Remdesivir had a reduced risk (OR 0.444, 95% CI 0.292–0.674) of self-reporting post-COVID-19 pain symptoms, particularly in the thorax/chest and lumbar spine.

### 4.1. General Effects of Remdesivir

The impact of Remdesivir on post-COVID-19 symptoms remains debated [13]. Boglione et al. [14] reported that the administration of Remdesivir during the acute phase of COVID-19 was associated with a lower risk (OR 0.641, 95% CI 0.413–0.782) of developing post-COVID-19 symptomatology six months post-hospitalization, though these authors did not specify which symptoms were affected. Conversely, Nevalainen et al. found no significant effect (RR 0.94, 95% CI 0.47–1.90) of Remdesivir on post-COVID-19 symptoms one year after hospitalization [16]. This study analyzed specific post-COVID-19 symptoms but did not find a significant impact on joint (OR 1.15, 95% CI 0.66–2.00) or muscle (OR 0.72, 95% CI 0.34–1.51) pain. Despite the lack of statistical significance, the study noted wide confidence intervals, suggesting potential benefits or harms [16]. One possible reason for these discrepancies is sample size differences, as Nevalainen et al. included 181 patients [16] whereas Boglione et al. [14] had 462 COVID-19 survivors, a sample size like our study, potentially making the former underpowered [16]. A recent study by Fésü et al. [17] also observed improvements in post-COVID-19 symptoms with Remdesivir administration but found no significant impact on functional outcomes.

While our findings suggest that the intravenous administration of Remdesivir during the acute COVID-19 phase may reduce the overall risk of post-COVID-19 pain, the distribution of pain symptoms was largely similar between groups, except for a lower prevalence of thorax/chest and lumbar spine pain in those who received antiviral treatment (Figure 1). Clinically, a subgroup of post-COVID-19 individuals develop widespread pain patterns resembling fibromyalgia syndrome [26]. Our study found no difference in the prevalence of widespread pain between groups, suggesting that intravenous Remdesivir does not influence this aspect. Given that this subgroup may exhibit nociplastic pain features [27], precision tailored treatment strategies should be considered [28]. As post-COVID-19 pain symptoms were self-reported, incorporating objective measures such as quantitative sensory testing could provide insights into pain phenotypes and sensitization mechanisms.

### 4.2. Mechanisms Explaining the Effects of Remdesivir

It has been proposed that antivirals may be effective for specific post-COVID-19 symptoms depending on their underlying mechanisms. Recent studies indicate that inflammatory pathways related to tissue damage play a role in some post-COVID-19 symptoms but not in others [29]. Our study suggests that the intravenous administration of Remdesivir at the acute COVID-19 phase serves as a protective factor against post-COVID-19 pain. Since antivirals inhibit SARS-CoV-2 replication [2], reduced viral replication may result in decreased pro-inflammatory response, leading to a lower immune response activation. Accordingly, antiviral treatment at the acute phase of SARS-CoV-2 infection may help to prevent post-COVID-19 symptoms linked to viral replication or heightened inflammatory responses. The fact that pain is strongly associated with inflammation [30], with studies suggesting that the presence of myalgia at the acute COVID-19 phase predicts post-COVID-19 pain [31], highlights the sensitivity of the muscular pain system to cytokine activity.

### 4.3. Previous Pain Conditions

Another key factor is the presence of chronic pain conditions before SARS-CoV-2 infection. A large retrospective study has identified that individuals with chronic overlapping pain conditions before infection had a 1.47-fold increased risk of long-COVID pain symptoms [32]. In our study, there was no significant difference in the prevalence of pre-existing chronic pain conditions between the Remdesivir and non-Remdesivir groups. Therefore, the lower incidence of post-COVID-19 pain in those COVID-19 survivors receiving antiviral treatment was not attributable to this factor. Nonetheless, due to the retrospective nature of medical data collection, this conclusion should be interpreted cautiously.

### 4.4. Antiviral Treatment and COVID-19 Severity

The use of antivirals during the acute COVID-19 phase has been linked to reduced mortality, shorter hospitalization duration, and lower disease severity [1]. Consequently, the protective effect of Remdesivir on post-COVID-19 pain could be attributed to a decrease in disease severity, leading to shorter hospital stay, decreased need for non-invasive ventilation, or lower ICU admission rates in this cohort. Boglione et al. suggested that the effect of Remdesivir on long-COVID could be due to these factors [14]. However, our study does not support this hypothesis, as ICU admission rates and hospitalization stay did not differ between the groups. The lack of differences in these parameters suggests that other factors contribute to the protective effect of Remdesivir. Nonetheless, we acknowledge that data on COVID-19 severity at hospitalization were not collected in our study, a limitation also noted by Fésü et al. [17].

### 4.5. Strengths and Limitations

The inclusion of a matched control group of COVID-19 survivors who did not receive the intravenous administration of Remdesivir and the long-term follow-up (18 months post-hospitalization) are strengths of the current study. However, several limitations must be acknowledged. First, our findings are applicable only to previously hospitalized COVID-19 survivors, as Remdesivir is administered intravenously, limiting its use in non-hospitalized populations. Additionally, all participants were recruited from four urban hospitals in Madrid, Spain, which may affect the generalizability of results to other regions and populations. Second, the cross-sectional design prevents an analysis of the longitudinal progression and fluctuating nature of post-COVID-19 pain. Retrospective data collection from medical records also limits causal interpretations. Furthermore, the absence of certain confounders, such as COVID-19 severity (e.g., severe pneumonia, saturation level) may influence findings, although no differences in ICU admission, hospitalization stay, or pre-existing conditions were observed. Prospective clinical trials could provide stronger causal evidence. Third, while the dosage of Remdesivir is standardized by the Food and Drug Administration (FDA) [33] and the European Medicines Agency (EMA) [34], it remains unknown whether different dosages might affect post-COVID-19 pain. Lastly, biological markers such as viral persistence, inflammatory mediators, and immune responses were not assessed, which could help clarify the mechanisms underlying Remdesivir’s effects. Future research should explore these factors to enhance the understanding of post-COVID-19 pain development and other long-term symptoms also present in people with post-COVID-19 condition.

## 5. Conclusions

This study found that COVID-19 survivors who received intravenous administration of Remdesivir during hospitalization developed lower post-COVID-19 pain symptomatology, particularly in the thorax/chest and lumbar spine. Further research is needed to determine whether oral antiviral treatments have similar effects on non-hospitalized populations to expand the applicability of these findings.

## Figures and Tables

**Table 1:** Pre- and post-COVID-19 pain symptoms according to administration or not of Remdesivir.

	No Remdesivir (*n* = 216)	Remdesivir (*n* = 216)	*p*-Value
Female *n* (%)	94 (43.5%)	94 (43.5%)	—
Age (years)	55.6 ± 12.7	55.4 ± 12.6	0.882
Weight (kg)	81.9 ± 15.2	80.8 ± 14.0	0.441
Height (cm)	167.4 ± 10.9	166.6 ± 9.2	0.409
Days at hospital (mean ± SD)	12.9 ± 12.6	16.6 ± 12.4	0.365
ICU admission *n* (%)	28 (12.9%)	29 (13.4%)	0.816
Pre-existing chronic pain conditions before infection			
Migraine	5 (2.3%)	1 (0.5%)	0.686
Headache	10 (4.6%)	10 (4.6%)	0.999
Arthritis	5 (2.3%)	2 (1.0%)	0.528
Arthrosis	23 (10.6%)	18 (8.3%)	0.434
Fibromyalgia	1 (0.5%)	1 (0.5%)	0.999
Localized musculoskeletal pain	42 (19.4%)	47 (21.8%)	0.596
Pre-existing medical conditions before infection			
Obesity (pre-existing)	20 (9.25%)	28 (13.0%)	0.248
Hypertension (pre-existing)	64 (29.6%)	51 (23.6%)	0.225
Diabetes (pre-existing)	25 (11.6%)	9 (4.2%)	0.242
Asthma (pre-existing)	10 (4.6%)	11 (5.1%)	0.827
COPD (pre-existing)	7 (3.25%)	6 (2.8%)	0.781
Cardiac diseases (pre-existing)	23 (10.65)	18 (8.3%)	0.435
Rheumatological diseases (pre-existing)	1 (0.45%)	0 (0.0%)	0.318
Location of post-COVID-19 pain symptomatology			
Generalized	18 (8.3%)	12 (5.5%)	0.273
Head	10 (4.6%)	9 (4.2%)	0.818
Cervical spine	4 (1.8%)	3 (1.4%)	0.705
Shoulder	4 (1.8%)	1 (0.5%)	0.178
Elbow–wrist	4 (1.8%)	1 (0.5%)	0.178
Hip	4 (1.8%)	2 (0.9%)	0.414
Knee	7 (3.3%)	3 (1.4%)	0.206
Thorax/chest	17 (7.8%)	5 (2.3%)	0.02 *
Lumbar	19 (8.8%)	7 (3.2%)	0.02 *
Lower extremity	24 (11.1%)	18 (8.3%)	0.354
Upper extremity	13 (6.0%)	6 (2.8%)	0.108

* Statistically significant differences between groups.

## Data Availability

Data are contained within the article.
